# That’s not my family: The undercover bipolar patient

**DOI:** 10.1192/j.eurpsy.2021.1647

**Published:** 2021-08-13

**Authors:** M.F. Tascon Guerra

**Affiliations:** Psychiatry, Hospital Ntra Sra del Prado, Talavera de la Reina, Toledo, Spain

**Keywords:** Capgras, bipolar disorder, OLANZAPINE, diagnosis

## Abstract

**Introduction:**

Bipolar disorder is a serious psychiatric condition based on depressive, manic, and mixed phases. Bipolar disorder has been usually divided into type I (manic phases and depressive phases) and type II (hypomanic and depressive phases). Furthermore, subsequent classifications have been developed based on subtypes complexity.

**Objectives:**

A case of a 52-year-old man is presented. The patient had suffered depressive symptoms and self-destructive ideation. He had sold his house to pay for drugs and prostitution, even though he was in quarantine for COVID-19. The patient had a history of depressive episodes with milder manic episodes which had been treated with antidepressants.

**Methods:**

Analytical and imaging tests were performed without findings. Mood stabilizing treatment was started based on lithium salts. He became perplexed and suspicious of the medication. Auditory hallucinations appeared congruent with the mood and he began to think that his relatives were dead; the patient started to communicate via telephone with actors. A treatment based on olanzapine 30mg/d was started.

**Results:**

The clinic was resolved with antipsychotic treatment. The diagnosis of Bipolar Disorder has been made in youth; however, some patients symptoms could be camouflaged and allow a functional life. Depressive episodes could present with psychotic symptoms and predispose more to suicide attempts than manic phases.

**Conclusions:**

The early-stage diagnosis should play a key role on bipolar disorder control. More public and clinic efforts are needed to prevent non-easily distinguishable cases which could derived on serious social and health problems.

**Disclosure:**

No significant relationships.

